# Novel sequence variants and a high frequency of recurrent polymorphisms in *BRCA1 *gene in Sri Lankan breast cancer patients and at risk individuals

**DOI:** 10.1186/1471-2407-8-214

**Published:** 2008-07-29

**Authors:** Wasanthi De Silva, Eric H Karunanayake, Kamani H Tennekoon, Marie Allen, Indrani Amarasinghe, Preethika Angunawala, Mohamed H Ziard

**Affiliations:** 1 Institute of Biochemistry Molecular Biology and Biotechnology, University of Colombo, 90, Cumaratunga Munidasa Mawatha, Colombo 03, Sri Lanka; 2 Department of Genetics and Pathology, Rudbeck Laboratory, University of Uppsala, Sweden; 3 National Cancer Institute, Maharagama, Sri Lanka; 4 Department of Pathology, Faculty of Medicine, Kynsey Road, Colombo 08, University of Colombo, Sri Lanka

## Abstract

**Background:**

Breast Cancer is the most commonly diagnosed cancer among Sri Lankan women. Germline mutations in the susceptibility genes *BRCA1 *and *BRCA2 *in hereditary breast/ovarian cancer, though low in prevalence, are highly penetrant and show geographical variations. There have been only a few reports from Asia on mutations in *BRCA1/2 *genes and none from Sri Lanka.

**Methods:**

A total of 130 patients with (N = 66) and without (N = 64) a family history of breast cancer, 70 unaffected individuals with a family history of breast cancer and 40 control subjects were analysed for *BRCA1 *mutations. All but exon 11 were screened by single strand conformation analysis (SSCP) and heteroduplex analysis. PCR products which showed abnormal patterns in SSCP were sequenced. Exon 11 was directly sequenced.

**Results:**

Nineteen sequence variants were found in *BRCA1 *gene. Two novel deleterious frame-shift mutations; c.3086delT/exon11 (in one patient) and c.5404delG/exon21 (in one patient and two of her family members) were identified. A possibly pathogenic novel missense mutation (c.856T>G/exon 11) and three novel intronic variants (IVS7+36C>T, IVS7+41C>T, IVS7+49del15) were characterised. Ten previously reported common polymorphisms and three previously reported intronic variants were also observed.

**Conclusion:**

After screening of 66 patients with family history and 64 sporadic breast cancer patients, 2 deleterious mutations (c.3086delT and c.5404delG) in two families were identified and two more possibly pathogenic mutations (c.856T>G and IVS17-2A>T) in two families were identified.

**Data base:**

*BRCA1 *- Gene Bank: Accession # U14680 Version # 14680.1

## Background

Breast Cancer is the commonest malignancy in women worldwide, accounting for 23% of all cancers and is surpassed only by lung cancer when cancers of both genders are considered [1]. In Sri Lanka, breast cancer is the most commonly diagnosed cancer among women, currently accounting for 25% of cases. However, Sri Lanka has a lower age standardized incidence rate of breast cancer (18.25) compared to other Asian countries (range 18.7 to 33.3) and to North America (99.4) [1,2].

A number of risk factors are known to affect the likelihood of developing breast cancer. Among them most potent are the inherited mutations in the breast cancer susceptibility (*BRCA*) gene 1 and 2 [3]. *BRCA1 *and *BRCA2 *mutations are highly penetrant with a lifetime risk of 46–87% and 26–84% respectively of developing breast cancer by the age of 70 years for mutation carriers [4-8]. *BRCA1 *is located in 17q21 and consists of 24 exons coding for 1863 amino acids [9]. *BRCA2 *(13q12.3) consists 27 exons coding for 3418 amino acids [10]. *BRCA1 *and *BRCA2 *tumor suppressor genes play a major role in DNA repair by homologous recombination, maintenance of chromosomal stability, activation of DNA damage checkpoints, transcription-coupled DNA repair, cell cycle regulation and ubiquitylation [11-13].

To date more than 1600 sequence variants of *BRCA1 *and 1800 sequence variants of *BRCA2 *have been described [14]. Some of these mutations were found only in specific ethnic groups and hence referred to as founder mutations. Due to these founder effects as well as due to other environmental and geographical factors, the prevalence of *BRCA1 *and *BRCA2 *mutations is variable among different populations [15]. Many studies from the developed world report the prevalence of *BRCA1 *and *BRCA2 *mutations of breast cancer to vary from 1.8 – 13.1% [16-19]. Prevalence of *BRCA1 *mutations in Asian countries vary from 0.8 to 8.6% [20-24]. In Sri Lanka, *BRCA *germ line mutations have not been previously characterised.

In the present study, 130 breast cancer patients, 70 at risk individuals and 40 control subjects were analysed for *BRCA1 *mutations. We report here 2 novel deleterious mutations, one novel possibly pathogenic mutation, three novel intronic variants, ten reported polymorphisms and three reported intronic variants.

## Methods

### Subjects

A total of 130 breast cancer patients (N = 66 with a family history of breast cancer and N = 64 sporadic breast cancer), 70 at risk individuals (those without disease but with a family history of breast cancer) and 40 healthy control subjects without a personal or a family history of any cancer (N = 20 females and N = 20 males) were studied. At risk individuals included 1, 2 or more first degree and second-degree relatives of one affected patient. This also included 5 first and 14 second degree relatives of one breast cancer patient diagnosed at less than 35 years of age. For 30 at risk individuals, their first and/or second-degree relative breast cancer patients were included in this study. Whereas for the rest, breast cancer patients had already deceased and no blood samples were available for analysis.

Mean age at diagnosis with breast cancer, number of family members affected with breast cancer or other cancers and ethnicity are summarised in Table 1. Thirty eight patients were below 40 years of age at diagnosis. Thirty three at risk individuals studied were below 40 years of age. Of the 66 patients with familial breast cancer, 44 had only one affected family member with 18 having an affected first degree relative and 26 having an affected second degree relative. Nineteen patients with familial breast cancer had 2 family members affected, and of these 5 had two affected first degree relatives, 11 had two affected second degree relatives and each of the remaining 3 patients had one first degree and one second degree relative with breast cancer. Three patients with familial breast cancer had one affected first degree relative and two affected second degree relatives each.

**Table 1:** Characteristics of patients and at risk individuals

	**Familial breast cancer patients ** **(N = 66)**	**Sporadic breast cancer patients ** **(N = 64)**	**At risk Individuals ** **(N = 70)**
Age (years) at diagnosis (patients)/sample collection (at risk individuals)			
Mean ± SD	46.97+9.05	47.37+10.06	40.51+12.01
≤ 40	18	20	33
> 40	48	44	37
Number of family members with breast cancer			
N = 0	0	64	0
N = 1	44 (1^st^: 18, 2^nd^: 26)	0	28
N = 2	19 (1^st^: 5, 2^nd^: 11, 1^st ^& 2^nd ^both: 3)	0	18
N = 3	03 (1^st ^and 2^nd^)	0	24
Number of family members with other cancers*			
N = 1	16	0	17
N = 2	07	0	24
N = 3	01	0	01
N = 4	01	0	0
Ethnicity			
Muslim	02	03	01
Sinhalese	62	58	68
Tamil	02	03	01

* colorectal, lung, oral, ovarian, thyroid, uterine cancers 1^st^: 1^st ^degree relative, 2^nd^: 2^nd ^degree relative affected with breast cancer

Majority of the patients, at risk individuals and controls were ethnically Sinhalese. There were no descendents of Europeans. The study had ethical approval from the Institution Review Board. All the study participants gave written informed consent to be included in the study. Patients were recruited from the National Cancer Institute, Maharagama and from those referred to Department of Pathology, Faculty of Medicine, University of Colombo. Unaffected individuals with a family history of breast cancer were from the Breast Clinic, National Cancer Institute, Maharagama. Socio-demographic and clinical data were obtained from the study participants and cancer diagnoses were confirmed by reviewing medical reports and pathology reports.

### Molecular studies

Genomic DNA was isolated from peripheral blood lymphocytes from 10 ml blood samples using the protocol described by Miller *et al *[25]. Specific primers were selected from BIC primer database for PCR amplification of all the exons of *BRCA1 *gene. The coding and neighbouring intronic regions were screened by a combination of single strand conformation analysis (SSCP) and heteroduplex analysis. Polymerase chain reaction (PCR) amplification was carried out in 25 μl volumes containing 50 ng of genomic DNA, 3.5 mmol/l MgCl_2_, 1× PCR buffer [10 mM Tris-HCl (pH 8.3), 50 mM KCl], 2.5 mmol/l dNTPs (Promega, Madison, WI, USA), 5 pmols of each primer and 0.5 U of *Taq *Polymerase (Promega). PCR reaction was carried out for 33 cycles and the thermal cycling conditions were denaturation at 94°C for 45 s, annealing at the respective optimal temperature for 45 s and extension at 72°C for 2 min. Optimal annealing temperature for different exons varied from 54°C to 60°C.

SSCP analysis was carried out by denaturing equal volumes of PCR product of the sample with denaturing loading buffer (95% formamide, 0.05% xylene cyanol, 0.05% bromophenol blue) heated at 95°C for 5 min. Denatured DNA were separated by polyacrylamide (10%) gel electrophoresis under non-denaturing conditions and run at 100 V for 8–10 h in 0.5 × TBE at 10–15°C). DNA fragments were visualized by silver staining [26]. Heteroduplex analysis was done by mixing equal volumes of denaturing loading buffer and a mixture of sample and normal control PCR products, denaturing at 95°C for 5 min and then allowing to re-anneal by incubating for 10 min at room temperature. The resulting double-stranded heteroduplex molecules were visualized by silver staining. Samples giving abnormal migration patterns on SSCP were reconfirmed using a second PCR product.

### DNA sequencing

Samples which showed an abnormal pattern on SSCP analysis were selected. DNA was eluted from the abnormal polyacrylamide gel bands and re-amplified using the same primers. PCR products were purified using GFX™ PCR DNA and Gel Band Purification Kit (GE Healthcare Bio-Sciences Corp, Piscataway, NJ, USA). Both strands were directly sequenced using a commercially available Cy™ Dye Termination Kit using the MegaBACE 1000 automated DNA sequencer (GE Healthcare). Mutations and sequence variants detected were reconfirmed by analysing a second sample and a second PCR product respectively. Exon 11 was analysed by direct sequencing. This analysis was limited to 51 patients with a family history of breast cancer, 22 at risk individuals (19 first and second degree relatives of a patient detected to have a novel deleterious mutation in exon 21 and 3 first degree relatives of a patient detected to have a novel missense mutation in exon 11 during the current study) and 22 controls.

## Results and discussion

Breast cancer incidence in Sri Lanka has increased during the last two decades. Mean age of onset at 47 years for familial and sporadic breast cancer patients in this study shows the importance of studying existence of genetic susceptibility in Sri Lankan breast cancer patients. A confirmative genetic test for *BRCA1 *or *BRCA2 *mutations can markedly reduce cancer risk by prophylactic mastectomy and oophorectomy [13].

This is the first report on *BRCA1 *mutations and polymorphisms in Sri Lankan breast cancer patients and at risk individuals. We have identified 19 sequence variants in *BRCA1 *gene (Tables 2 and 3). Two novel deleterious frame-shift mutations, c.3086delT in exon 11 [BIC: AC:17840] and c.5404delG in exon 21 [BIC: 17841], and one novel missense possibly pathogenic mutation, c.856T>G in exon 11 [BIC: AC17839] were detected (Table 2). Frame-shift mutation c.3086delT in exon 11 was observed in one breast cancer patient and c.5404delG in exon 21 in one breast cancer patient, one of her first degree and one second degree relative. Possibly pathogenic, novel missense mutation c.856T>G in exon 11 was seen in one breast cancer patient. Three novel intronic variants (IVS7+36C>T, IVS7+41C>T, IVS7+49del15) were found in exon 7 (Table 3) [BIC: AC17836, BIC: AC17837, BIC: AC17838 respectively]. Ten previously reported polymorphisms in exons 11 [BIC: AC17844, BIC: AC17845, BIC: AC17846, BIC: AC17847, BIC: AC17848, BIC: AC17849], 13 [BIC: AC17850] and 16 [BIC: AC17851, BIC: AC17852, BIC: AC17853] and one reported intronic variant each in exons 7 [BIC: AC17842], 8 [BIC: AC17843] and 18 [BIC: AC17854] were identified (Table 3).

**Table 2:** Clearly pathogenic and possibly pathogenic *BRCA1 *mutations identified

**E/I**	**NT**	**Base Change**	**Codon**	**AA Change**	**Designation**	**Variation type**	**BIC Entry**
11-A	856	T>G	246	Leu>TrP	c.856T>G	M-UV	No
11-C	3086	delT	989	Stop999	c.3086delT	F	No
I-18	5194-2	A>T	Non- coding	--	IVS17-2A>T	IVS	Yes
21	5404	delG	1762	Stop1764	c.5404delG	F	No

E/I: Exon/Intron, F: frame shift, M: missense alteration, UV: unclassified variant

**Table 3:** Common polymorphisms and intronic variants of *BRCA 1 *gene identified

**E/I**	**NT**	**Base Change**	**Codon**	**AA Change**	**Designation**	**Variation type**	**BIC Entry**
I-7	560+36	C>T	Non- coding	--	IVS7+36T>C	UV	No
I-7	560+38	T>C	Non- coding	--	IVS7+38T>C	UV	Yes
I-7	560+41	C>T	Non- coding	--	IVS7+41C>T	UV	No
I-7	560+49	DelT	Non- coding	--	IVS7+49del15	UV	No
I-8	561-34	C>T	Non- coding	--	IVS7-34C>T	IVS	Yes
11-B	2196	G>A	693	Asp > Asn	c.2196G>A	M-P	Yes
11-B	2201	C>T	694	Ser>Ser	c.2201C>T	Silent-P	Yes
11-B	2430	T>C	771	Leu>Leu	c.2430T>C	Silent-P	Yes
11-C	2731	C>T	871	Pro>Leu	c.2731C>T	M-P	Yes
11-C	3232	A>G	1038	Glu>Gly	c.3232A>G	M-P	Yes
11-D	3667	A>G	1183	Lys>Arg	c.3667A>G	M-P	Yes
13	4427	T>C	1436	Ser>Ser	c.4427T>C	Silent-P	Yes
16	4931	A>G	1604	Gln>Gln	c.4931A>G	Silent-P	Yes
16	4956	A>G	1613	Ser>Gly	c.4956A>G	M-P	Yes
16	5075	A>G	1652	Met>Ile	c.5075G>A	M-P	Yes

E/I: Exon/Intron, M: missense alteration, P: polymorphism, UV: unclassified variant

**Figure 1:**
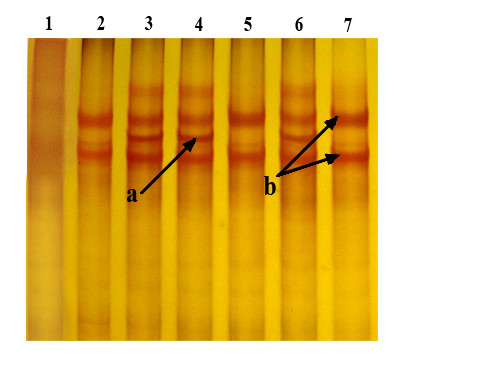
**SSCP analysis of PCR products of exon 21/*BRCA1***. Lane 3, 4 and 6 are samples with the c.5404delG mutation. Lane 2, 5 and 7 are wild type. Lane 1 is a non template control. a: additional band seen in the presence of mutation, b: banding pattern shown by wild type.

**Figure 2:**
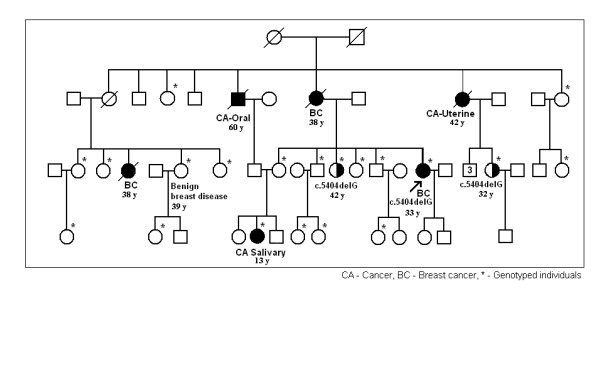
**Pedigree of family F-01**. Age of onset of the disease for members affected with breast cancer, other cancers and benign breast disease indicated.

SSCP analysis showed abnormal migration patterns in polyacrylamide gels for exons 7, 8, 13, 16, 18 and 21. Abnormal migration patterns in SSCP analysis of exon 21 were seen only in three study participants (III.12, III.16, III.19) of a single sib ship (Figure 1). The pedigree of this family (F-01) is shown in Figure 2. Direct sequencing showed the presence of a novel frame shift mutation: c.5404delG (codon 1762) resulting in a stop codon at 1764 of *BRCA1 *protein in all three of them. This family was Sinhalese in ethnicity and had five individuals of three different generations affected by breast, uterine and salivary gland cancer. Out of the three with the mutation, one (III.16) developed breast cancer at 33 years of age and the other two were her first and second-degree female relatives. However these two family members do not have clinical or radiological evidence of breast cancer to date. The patient and her sister carrying the same mutation were the daughters of a breast cancer patient diagnosed with the disease at 38 years of age. The second-degree female relative who carries the same mutation was the daughter of our breast cancer patient's maternal aunt who was diagnosed with uterine cancer at the age of 42 years. These breast and uterine cancer patients in the FII generations had deceased and no archival specimens were available for genetic studies. We screened 19 first and second degree living relatives of our index case (III.16) including her three sisters and two brothers for *BRCA1 *mutations.

Mutation c.5404delG has not been previously reported in the breast cancer information core [14]. It is present in the area of two BRCT domains (a.a 1646–1859) of *BRCA1 *protein. BRCT domain is a unique feature of proteins which are involved in DNA repair and check point control [13]. One study has shown that even a *BRCA1*-BRCT missense mutation created a conformational change and functional instability in *BRCA1 *protein [27]. According to the BIC database, up to date only twelve exonic and nine intronic variations have been identified in exon 21/*BRCA1 *[14]. None of the Asian countries reported mutations in exon 21 and a very few are reported from other countries [28].

Deleterious frame shift mutation, c.3086delT in exon 11 was identified in one patient who developed breast cancer at the age of 46 years. Her maternal grandmother who is now deceased was diagnosed with breast cancer at 52 years of age. This frame shift mutation creates a stop codon at 999 in *BRCA1 *protein and is located in the area of DNA binding domain of *BRCA1*, which spans amino acids 452 to1079. RAD51 binds to this area and it is a key component of the DNA damage repair by homologous recombination [13]. This mutation is therefore likely to be of functional significance and further studies will be needed to clarify its role.

One possibly pathogenic novel missense alteration c.856T>G (exon 11) was found in a breast cancer patient diagnosed at 45 years of age with a family history of breast and uterine cancer. Her mother and a sister both of whom are now deceased were diagnosed with breast cancer at the age of 50 and 35 years respectively and a maternal aunt was diagnosed with uterine cancer at 52 years of age. This missense alteration converts amino acid leucine to tryptophan at codon 246 of *BRCA1*. Both are nonpolar and hydrophobic but the former is an aliphatic and the latter an aromatic amino acid. Thus conformational changes in the protein are possible. Three healthy first degree relatives of this patient who were included in this study did not carry c.856T>G mutation.

**Figure 3:**
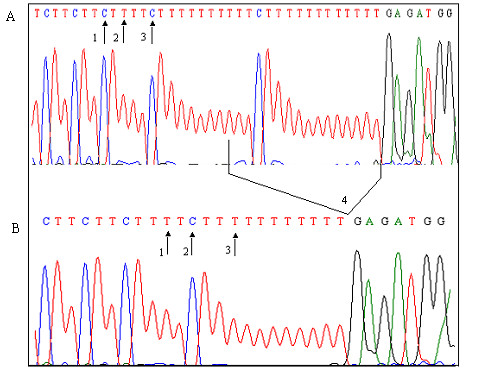
**Sequencing graphs of 4 intronic variants**. **A **– Normal control, **B **– Mutant: IVS7+36C>T (1), IVS7+38T>C (2), IVS7+41C>T (3) and IVS7+49del15 (4).

We identified 4 co-existing intronic variants IVS7+36C>T, IVS7+38T>C, IVS7+41C>T and IVS7+49del15 in intron 7. Except IVS7+38T>C, other three variants have not been previously reported in BIC database. SSCP analysis showed very clear banding patterns in normal, heterozygous and homozygous individuals and sequencing of eluted DNA confirmed the presence of 4 intronic variants (Figure 3). 51 familial breast cancer patients, 55 sporadic breast cancer patients, 61 at risk individuals and 28 control subjects had these 4 co-existing intronic variants with an allele frequency of 0.48, 0.62, 0.60 and 0.54 respectively. IVS7+38T>C has been previously reported from India but only in one sporadic breast cancer patient [24].

We found one previously reported intronic variant each in introns 8 and 18. IVS7-34C>T in exon 8 was found in two patients with a family history of breast cancer. One of them has two first degree relatives (mother and one sister) and the other has two second degree relatives (maternal grandmother and maternal aunt) affected with breast cancer. This variant has also been reported in Indian breast cancer patients and there were 8 records of the same sequence variant in BIC database. It does not appear to show any clinical importance [14].

IVS17-2A>T in intron18 was found in a patient diagnosed with breast cancer at the age of 35 years. Her mother who is now deceased was diagnosed with uterine cancer and mother's sister and her daughter were diagnosed with breast cancer at the age of 55 and 32 years respectively. According to BIC database this intronic variation located at the splice-site of the intron is clinically important. Nearly 5% of all *BRCA1 *and *BRCA2 *sequence alterations are splice-site mutations (BIC database, 2007), but very few studies have been carried out to identify the pathogenic importance of these [29-31].

Common polymorphisms in *BRCA1 *gene appear to be highly prevalent in Sri Lankan breast cancer patients and in healthy controls. In this study, we found ten previously reported polymorphisms c.2196G>A, c.2201C>T, c.2430T>C, c.2731C>T, c.3232A>G, c.3667A>G in exon 11, c.4427T>C in exon 13 and c.4931A>G, c.4956A>G, c.5075G>A in exon 16. The allele frequencies of some of the polymorphisms observed by us were greater than what has been reported by others (ie: allele frequencies of 0.35 or greater for c.2201C>T, c.2430T>C, c.3232A>G, c.3667A>G, 0.52 or greater for c.4427T>C and 0.49 or greater for c.4956A>G) [16,19,21,23,32-34]. However, there was no discernible difference in these allele frequencies between familial breast cancer, sporadic breast cancer, at risk individuals and healthy controls in the present study (data not shown).

Both c.4931A>G and c.4956A>G polymorphisms of exon 16 co-existed in three unrelated breast cancer patients with a family history of breast cancer. Polymorphisms c.5075G>A and c.4956A>G of exon 16 co-existed in 6 breast cancer patients, two at risk individuals and two controls all of whom were un related. Co-existence of c.4956A>G with c.4931A>G has not been reported previously. Although both c.4956A>G and c.5075G>A have been reported from India and Italy, whether these co-existed in the same individual has not been indicated [19,24]. Other studies report a prevalence rate between 0.2% and 7% for c.5075G>A polymorphism [16,19,34]

We also identified a reported missense polymorphism c.2196G>A in exon 11 of *BRCA1 *in a second degree maternal relative (III.6) of our index case in family F-01. She developed a benign breast tumour which was removed by lumpectomy recently and also suffers from a number of medical conditions namely, non-insulin dependent diabetes mellitus, chronic obstructive airway disorder, cervical spondylosis and hypertension. Her sister died of breast cancer at the age of 38 years. This particular polymorphism which converts Asp (negatively charged) to Asn (polar uncharged) has been frequently reported in different populations for example from Saudi Arabia, Greece and Belgium [17,34,35].

Out of 20 members of F-01 family included in this study one female member (IV.12) had no polymorphisms or intronic variants whereas all other 19 members including the breast cancer patient and two family members with the exon 21 mutation had all six common polymorphisms and intron 7 variations reported in the present study. The fact that one person with the exon 21 mutation developed breast cancer and the other two with the exon 21 mutation are yet free from the disease suggest that the six common polymorphisms and the intron 7 variations are unlikely to predispose to or protect from the disease in exon 21 mutation carriers.

## Conclusion

We identified 19 sequence variants in *BRCA1 *gene by screening 66 familial and 64 sporadic breast cancer patients, 70 at risk individuals and 40 control subjects. Six of them were novel sequence variants. Two novel clearly pathogenic (c.3086delT and c.5404delG) and 2 possibly pathogenic [c.856T>G (novel) and IVS17-2A>T (reported)] mutations were identified. Three novel un-classified intronic variants; IVS7+36C>T, IVS7+41C>T, IVS7+49del15, twelve common polymorphisms in exons 11, 13 and 16 and two common intronic variants in I- 7 and I-8 of *BRCA1 *gene were also identified. Prevalence of *BRCA1 *mutations in our study was 6.25% (4/64) among familial breast cancer patients. Co-existence of polymorphisms c.4956A>G and c.4931A>G in exon 16 of *BRCA1 *gene and co-existence of IVS7+36C>T, IVS7+38T>C, IVS7+41C>T, IVS7+49del15 in I-7 of *BRCA1 *gene, have not been previously reported. This study should be extended to analyse a larger number of patients to identify other pathogenic mutations in the *BRCA 1 *gene as well as mutations in the *BRCA 2 *gene.

## Competing interests

The authors declare that they have no competing interests.

## Authors' contributions

WDS helped acquisition of samples, carried out molecular genetic studies, sequence alignment and drafted the manuscript. EHK conceived and designed the study, helped molecular genetic studies, data analysis and revision of the manuscript. KHT helped designing and coordinated the study, assisted clinical data and sample collection, molecular genetic studies, data analysis and revision of the manuscript. MA helped molecular genetic studies. IA and PA provided clinical expertise, recruitment of study participants and supervised clinical data and sample collection. MHZ collected clinical data and samples form the F I family and assisted molecular genetic studies. All authors read and approved the final manuscript.

## Pre-publication history

The pre-publication history for this paper can be accessed here:


